# Intraosseous Meningioma of Spheno-Orbital Localization: A Case Report

**DOI:** 10.7759/cureus.37970

**Published:** 2023-04-22

**Authors:** Keita Ansoumane Hawa, Coulibaly Moussa, Meriam Benzalim, ALJ Soumaya

**Affiliations:** 1 Department of Radiology, Ibn Tofail University Hospital, Marrakech, MAR; 2 Faculty of Medicine and Pharmacy, Cadi Ayyad University, Marrakech, MAR

**Keywords:** benign, tumor, bone, brain, meningeum

## Abstract

Intracranial meningiomas are benign extra-axial brain tumors. Their etiology is undetermined and several hypotheses have been proposed to explain their genesis. The clinical symptomatology of intracranial meningiomas is atypical and varies according to the location of the lesion, its size, and its relationship with neighboring organs. Imaging is essential for establishing a positive diagnosis, but the way to a diagnosis of certainty remains histological. In this article, we describe the CT and magnetic resonance imaging aspects of an intraosseous meningioma discovered in a patient in her forties that presented with right proptosis, and whose brain MRI revealed a cranial lesion with adjacent meningeal involvement; the CT scan subsequently performed allowed a better analysis of the bone lesion, the appearance of which suggested an intraosseous meningioma. This diagnosis was confirmed by a histological exam. The purpose of this article is to illustrate the CT and MRI aspects of this entity by reporting a case of intraosseous meningioma of spheno-orbital location.

## Introduction

Intracranial meningiomas are benign extra-axial brain tumors that represent up to 53% of benign intracranial lesions and 37.6% of all brain pathologies [1], making them the second most frequent intracranial tumor after gliomas. Women are the most affected with a ratio of 2:3 according to different studies. The intraosseous development of meningiomas is a rare ectopic localization that constitutes only 1 to 2% of all intracranial meningiomas [2]. Its etiology is unknown and several hypotheses have been proposed to explain its genesis. Its clinical symptomatology is atypical, the spheno-orbital involvement, for example, can lead to progressive, painless exophthalmos with a gradual decrease in visual acuity, while neurological signs can be observed in intraosseous meningiomas with extension to the internal table. Imaging has a crucial role in the establishment of a positive diagnosis, highlighting the different types of presentation of intraosseous meningiomas. In this article, we present the CT and MRI findings of a spheno-temporo-orbital intraosseous meningioma.

## Case presentation

**Figure 1:**
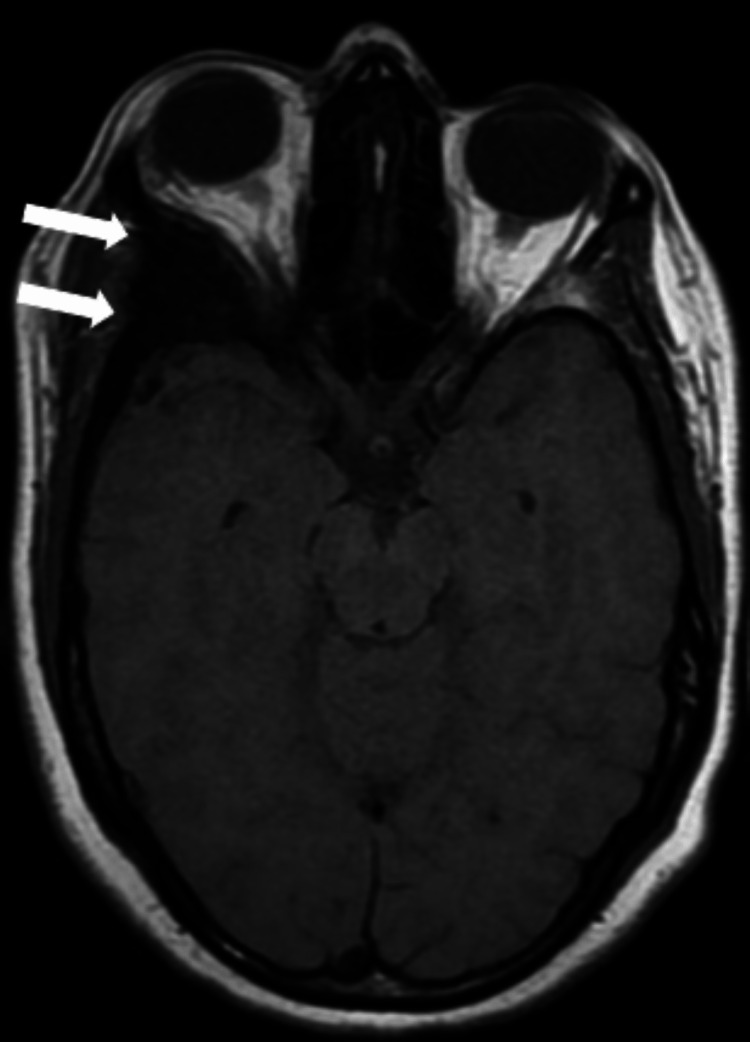
Brain MRI axial sequence Right spheno-fronto-temporo-orbital intraosseous lesion in hyposignal on the T1 sequence (white arrows).

**Figure 2:**
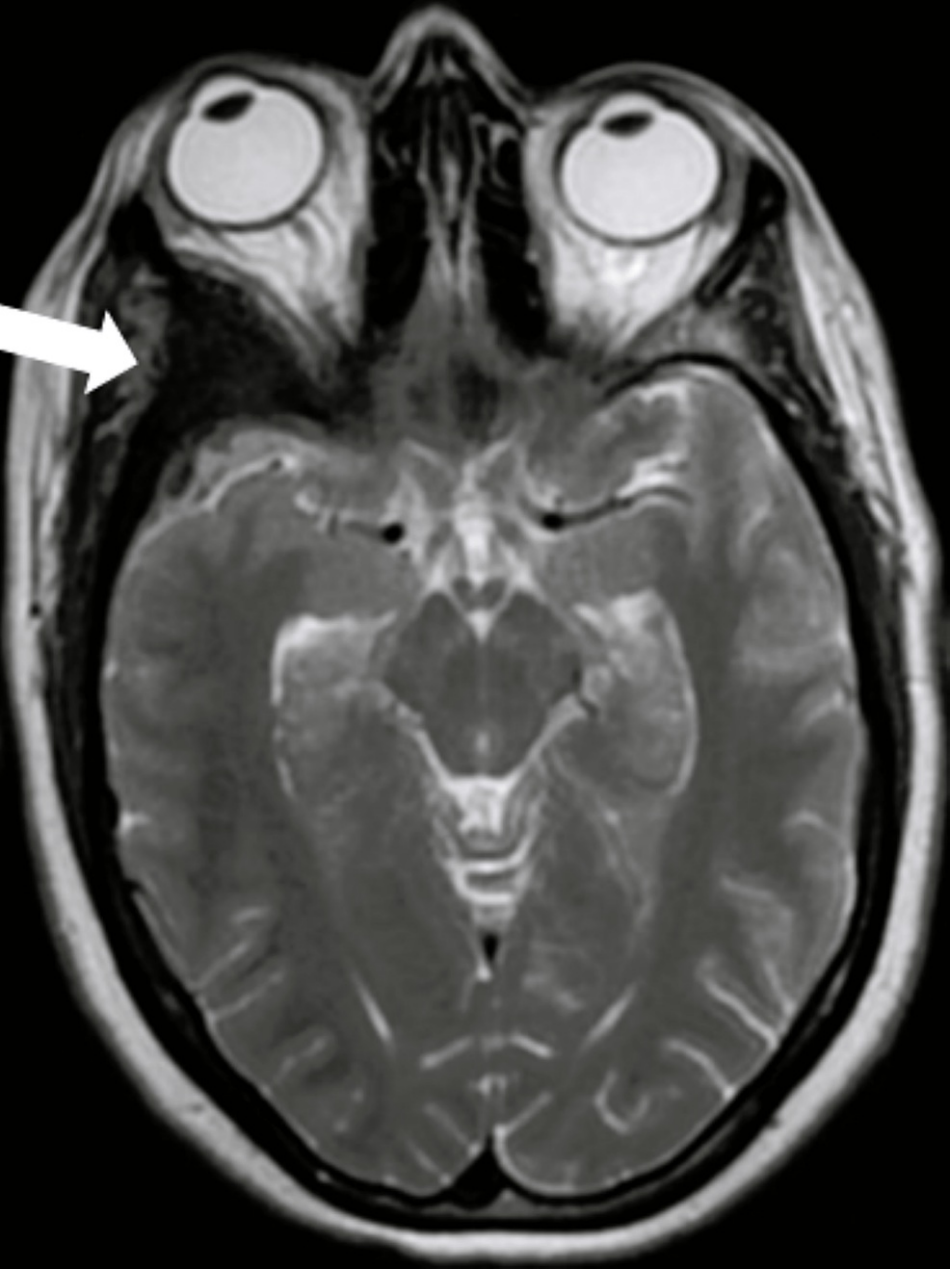
Brain MRI axial sequence Right spheno-fronto-temporo-orbital intraosseous lesion in hyposignal on the T2 sequence (white arrow).

**Figure 3:**
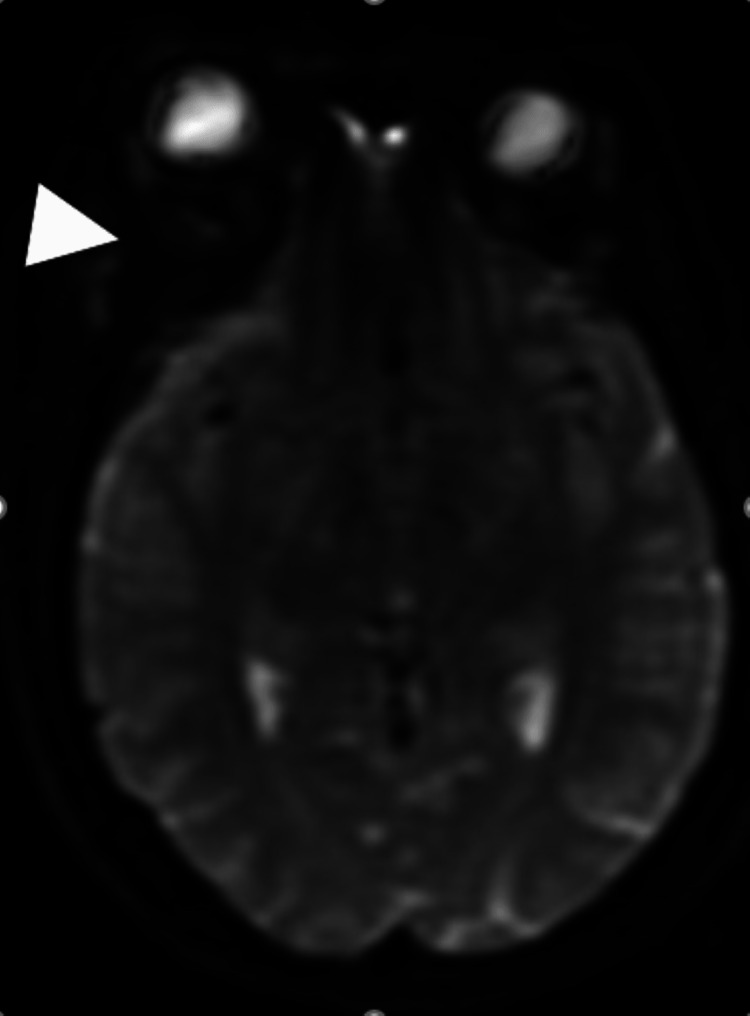
Brain MRI axial sequence Right spheno-fronto-temporo-orbital intraosseous lesion in hyposignal on the diffusion sequence (arrowhead).

**Figure 4:**
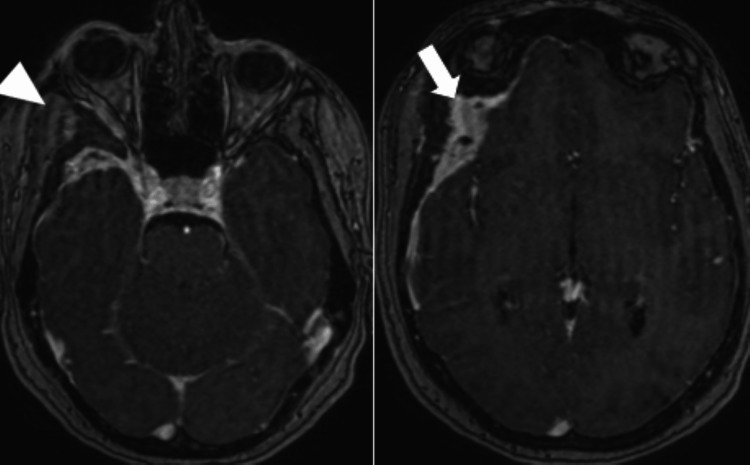
Brain MRI axial sequence Right spheno-fronto-temporo-orbital mass (arrowhead) with thickening and meningeal enhancement opposite (arrow).

**Figure 5:**
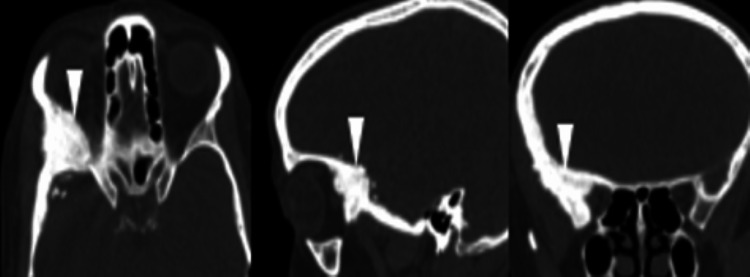
Brain scan Cerebral CT bone window in axial, sagittal, and coronal sections: spheno-orbital osteocondensing lesion with loss of right cotico-spongiosa differentiation.

A 42-year-old woman, with no particular pathological or traumatic history, presented with progressive headaches evolving for 28 months, dizziness, photophobia, and right proptosis. The clinical examination of the patient revealed a non-axial right unilateral exophthalmos, and on palpation, a hard and immobile right temporal swelling was noted. There was also a decrease in visual acuity, and the fundoscopy showed papilledema. The contralateral eye showed no abnormality. MRI revealed a right intraosseous spheno-fronto-temporo-orbital lesional process in hyposignal on the T1, T2, and diffusion sequences, and enhancing after contrast administration. It was associated with the thickening and enhancement of the adjacent meninges after gadolinium injection (Figure 1-4). The cerebral scanner carried out in addition allowed better analysis of the bone structures, making it possible to objectify on the bone window a right spheno-orbital osteocondensation with cortico-spongy dedifferentiation (diploe-tables), and spiculated aspect of the internal and external tables extended to the ipsilateral frontal and temporal bones, associated with meningeal thickening and enhancement (Figure 5). The patient underwent surgical management, with pathological confirmation of the diagnosis of intraosseous meningioma.

## Discussion

Meningiomas are the most common primary intracranial tumors. They arise from the meningothelial cells of the arachnoid membrane and develop in the subdural space [2]. They represent approximately 53% of benign intracranial lesions and 37.6% of all brain pathologies [1]; This makes it the second most frequently observed intracranial tumor after glioma. Forms evolving outside their usual location are called ectopic [3] and are generally found in the sinuses of the face, the nasal cavity, the orbits, the salivary glands, the nasopharynx, and the cranial vault [4]. The brain region is by far the preferred site, with 68% of primary extradural meningiomas [5]. Intraosseous localizations are generally found in the frontoparietal and sphenorbital regions [3, 5, 6], as was the case in our patient. This type of meningioma remains rare, with an incidence of 1% to 2% of all these tumors [3, 5, 6,7]. This pathology often occurs in the last decade of life, women are the most affected with a ratio of 2:3 according to the studies.

Clinically, the symptomatology is not typical, as it varies according to the location of the lesion, its size, and its relationship with neighboring organs [4], and manifests in the sphenorbital region with progressive, painless exophthalmos with a gradual decrease in visual acuity leading to blindness without associated neurological manifestations in some cases.

Involvement of the oculomotor nerve or the trigeminal nerve is not exceptional [2, 4]. Neurological signs can be observed in intraosseous meningiomas presenting an extension to the internal table with a mass effect on the cerebral parenchyma [8, 9]. This corresponds with the clinical data of our patient. It can also be demonstrated as a palpable mass in the temporal region. Imaging plays an essential role in diagnosis, especially CT and MRI. Standard radiography has a limited contribution to the diagnosis of spheno-orbital intraosseous meningiomas due to the anatomy of the cranial bone which is superimposed [3, 6, 8]. Computed tomography plays an essential role in the diagnosis by allowing the analysis of the tumor and its osseous extension to the parenchyma and soft tissues [9]. Up to 59% of patients present with osteocondensation-type lesions, while lytic lesions are encountered in 36% of cases; only 6% of patients present with mixed lesions [3,6]. Bone window reconstructions are necessary to evaluate lytic or hyperostotic bone reactions in order to discern the different bone structures affected (diploe, internal table, and external table) and study the aspect of hyperostosis which can be spiculated or smooth. The spicules of intraosseous meningiomas have a stocky and regular appearance that should not be confused with the fine and anarchic appearance of the spicules of malignant tumors [10].

Condensing lesions do not often enhance in post-contrast. We must systematically look for the thickening and enhancement of the dura mater. Magnetic resonance imaging allows a better analysis of the anatomical structures by showing their different components. The tumor appears in marked hyposignal on T1- and T2-weighted sequences. The intravenous injection of gadolinium will increase contrast uptake within the intraosseous component and at the level of the adjacent meningeal envelopes. It allows for the assessment of meningeal extension which is often underestimated by the scanner because of its low differential contrast between the bone and the enhanced meninges, thus making it possible to avoid a recurrence by partial excision. MRI can also detect intracranial extension [11]. Scintigraphy reveals hyperuptake in most cases [12].

The differential diagnosis depends on the radiological presentation. With hyperostotic lesions, the differential diagnosis is as follows: sclerotic hemangiomas which are well-defined lesions, with honeycomb trabeculations associated with a periosteal reaction which on MRI appears in heterogeneous T1 hypersignal; fibrous dysplasias appear as ground glass with sharp outlines; the osteoma shows regular and clear contours; and finally osteoblastic metastases and Paget's disease. As for lytic lesions, the differential diagnosis is as follows: osteosarcoma which presents fine and anarchic spicules; osteolytic metastases, and myeloma lacuna. In young subjects, eosinophilic granulomas and epidermoid cysts should also be discussed [2, 4].

Therapeutic management requires surgery by performing a fronto-temporo-sphenoidal craniectomy with resection of all the infiltrated tissues. In the event of incomplete resection, additional radiotherapy may be recommended [13]. Tumor remnants have a slow evolution, thus requiring annual monitoring by MRI, which allows the detection of tumor recurrence. Correction of visual disturbances postoperatively can be a valuable indicator of this evolution [14, 15].

## Conclusions

Intraosseous meningiomas are infrequent benign tumors of ectopic location and increasing evolution. The clinical symptomatology is dominated by ophthalmological manifestations, especially in the temporo-spheno-orbital forms. Imaging is nowadays, the first means of diagnosis because it makes it possible to confirm or disprove the existence of a bone lesion and to analyze the radiological characteristics of the lesion. However, the gold-standard for a diagnosis of certainty remains histological.
